# Hypoxia-induced downregulation of microRNA-186-5p in endothelial cells promotes non-small cell lung cancer angiogenesis by upregulating protein kinase C alpha

**DOI:** 10.1016/j.omtn.2023.01.015

**Published:** 2023-01-27

**Authors:** Vivien Becker, Xu Yuan, Anne S. Boewe, Emmanuel Ampofo, Elke Ebert, Johannes Hohneck, Rainer M. Bohle, Eckart Meese, Yingjun Zhao, Michael D. Menger, Matthias W. Laschke, Yuan Gu

**Affiliations:** 1 Institute for Clinical & Experimental Surgery, Saarland University, 66421 Homburg/Saar, Germany; 2 Fudan University Shanghai Cancer Center and Institutes of Biomedical Sciences, Department of Oncology, Shanghai Medical College, Fudan University, Shanghai 200032, China; 3 Institute of Pathology, Medical Center, Saarland University, 66421 Homburg/Saar, Germany; 4 Institute of Human Genetics, Saarland University, 66421 Homburg/Saar, Germany

**Keywords:** MT: non-coding RNAs, endothelial cell, hypoxia, miR-186, NSCLC angiogenesis, PKCα, ERK

## Abstract

The tumor microenvironment stimulates the angiogenic activity of endothelial cells (ECs) to facilitate tumor vascularization, growth, and metastasis. The involvement of microRNA-186-5p (miR-186) in regulating the aberrant activity of tumor-associated ECs has so far not been clarified. In the present study, we demonstrated that miR-186 is significantly downregulated in ECs microdissected from human non-small cell lung cancer (NSCLC) tissues compared with matched non-malignant lung tissues. *In vitro* analyses of primary human dermal microvascular ECs (HDMECs) exposed to different stimuli indicated that this miR-186 downregulation is triggered by hypoxia via activation of hypoxia-inducible factor 1 alpha (HIF1α). Transfection of HDMECs with miR-186 mimic (miR-186m) significantly inhibited their proliferation, migration, tube formation, and spheroid sprouting. In contrast, miR-186 inhibitor (miR-186i) exerted pro-angiogenic effects. *In vivo*, endothelial miR-186 overexpression inhibited the vascularization of Matrigel plugs and the initial growth of tumors composed of NSCLC cells (NCI-H460) and HDMECs. Mechanistic analyses revealed that the gene encoding for protein kinase C alpha (PKCα) is a *bona fide* target of miR-186. Activation of this kinase significantly reversed the miR-186m-repressed angiogenic activity of HDMECs. These findings indicate that downregulation of miR-186 in ECs mediates hypoxia-stimulated NSCLC angiogenesis by upregulating PKCα.

## Graphical abstract

**Figure:**
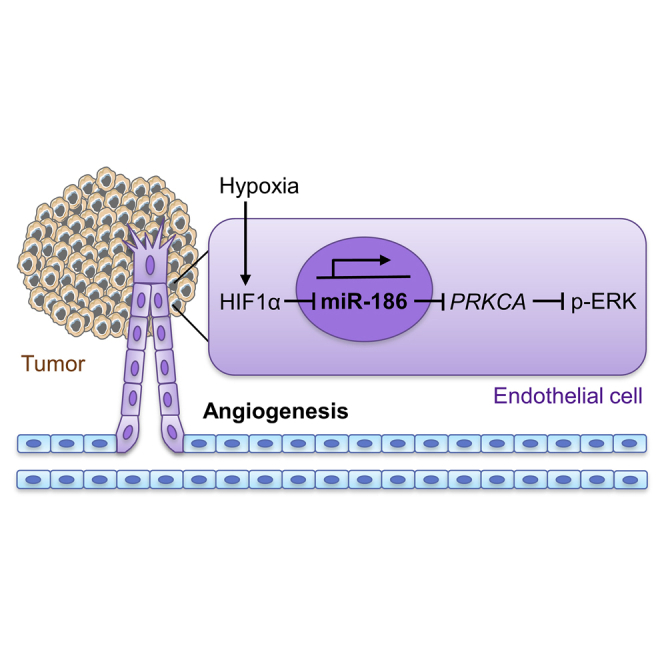

## Teaser

The present study demonstrates that miR-186 is downregulated in tumor endothelial cells of human NSCLC patients. This endothelial miRNA potently inhibits multiple mechanisms of blood vessel formation via directly targeting protein kinase C alpha. Accordingly, endothelial miR-186 represents a potential therapeutic target for the future anti-angiogenic treatment of NSCLC.

## Introduction

Tumor growth and metastasis crucially rely on angiogenesis, which is defined as the development of new blood vessels from pre-existing ones.^1^ In fact, once a tumor exceeds 1–2 mm^3^, simple diffusion processes can no longer sufficiently supply the tumor tissue with oxygen.^2^ Hypoxia drives the tumor cells to release various pro-angiogenic factors into the surrounding tumor microenvironment.^3^ Endothelial cells (ECs) are activated by these factors to degrade the vascular basement membrane, proliferate, migrate, and assemble into a new vascular network in the tumor.^4^ Compared with the blood vessels within normal tissues, tumor vessels are highly irregular and leaky, resulting in hypoxic regions even in highly vascularized tumors.^5^ The hypoxic tumor microenvironment further accelerates tumor angiogenesis by upregulating multiple pro-angiogenic pathways in ECs mainly via activation of the transcriptional factor hypoxia-inducible factor 1 alpha (HIF1α).^5^ Given the fact that angiogenesis promotes tumor progression and metastasis, the inhibition of tumor angiogenesis is a promising strategy for cancer therapy.^6^

MicroRNAs (miRNAs) serve as important regulators of EC angiogenesis. They are endogenous, single-stranded, and non-coding RNA molecules with a length of 19–23 nucleotides.^7^ They are estimated to modulate over 60% of protein-coding messenger RNAs (mRNAs) in mammalian cells, primarily through binding to the 3′ untranslated region (UTR) of mRNA, resulting in the downregulation of target gene.^8^ Each miRNA has the potential to regulate many target genes, allowing for high combinatorial complexity and regulatory potency.^9^

Numerous studies have suggested that microRNA-186-5p (miR-186) plays a pivotal role in tumor cell apoptosis, migration, invasion, and drug resistance.^10^ Its dysregulation in body fluids and tumor tissues of cancer patients makes this miRNA a promising biomarker for the diagnosis and prognosis of different cancer types, including non-small cell lung cancer (NSCLC).^10^^,^^11^ In addition, miR-186, which is located on chromosome 1q31.1, is highly conserved among mammals^11^ and enriched in different types of ECs.^12^ However, the regulation and function of miR-186 in ECs during tumor angiogenesis still need to be elucidated.

In the present study, we compared the expression of miR-186 in ECs dissected from human NSCLC tissues and adjacent non-tumor tissues. We then analyzed the effects of miR-186 on the development of new microvessels in a panel of *in vitro* and *in vivo* assays. Moreover, we performed a mouse flank tumor model to evaluate the effects of endothelial miR-186 on NSCLC angiogenesis and growth. Finally, we identified the functional target of miR-186 that mediates its anti-angiogenic function.

## Results

### Expression of miR-186 in ECs of NSCLC patients

**Figure 1:**
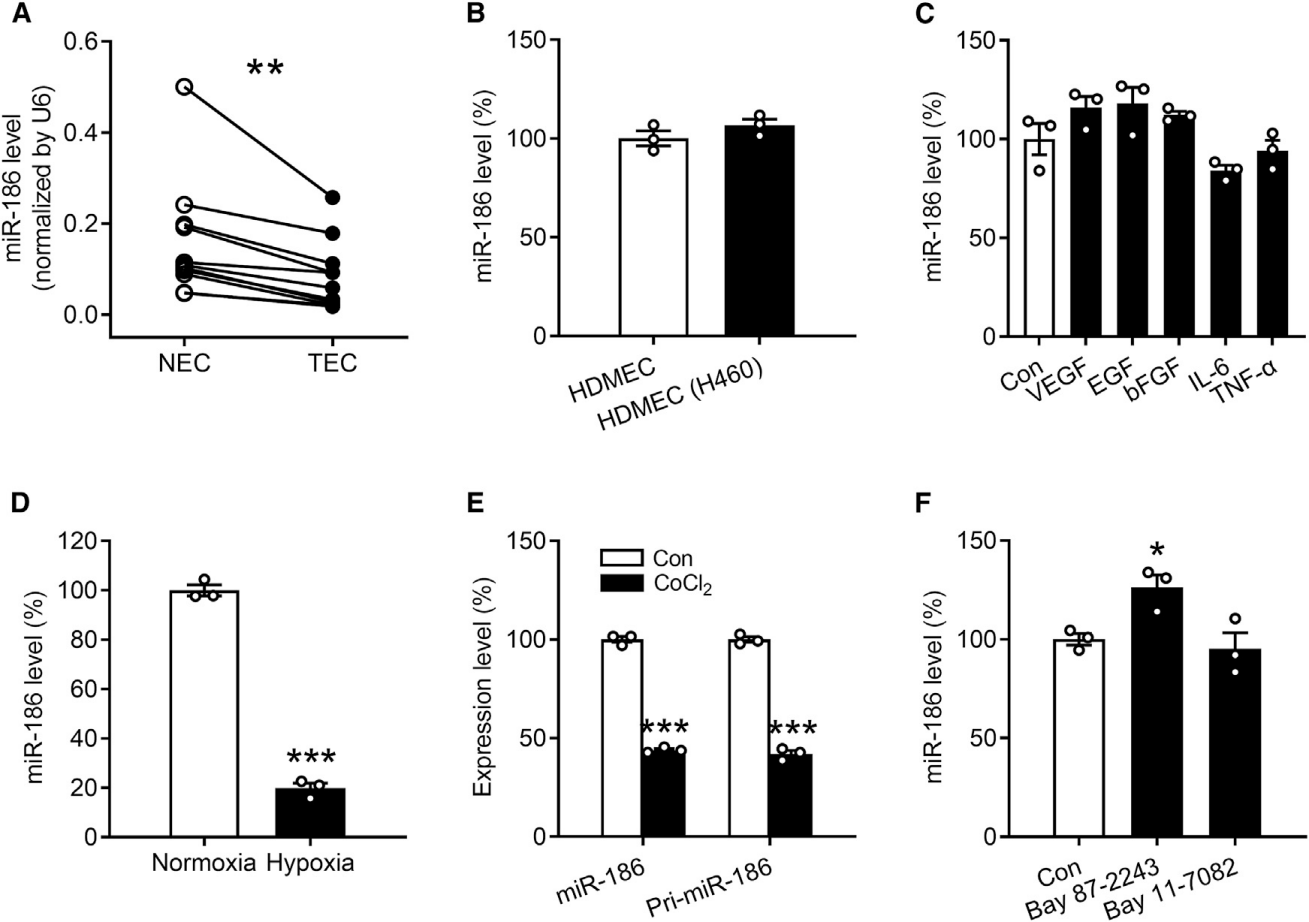
Hypoxia downregulates miR-186 transcription in ECs (A) miR-186 level (normalized by U6) in ECs dissected from non-tumor (NECs) or tumor tissues (TECs) of NSCLC patients by means of LCM, as assessed by qRT-PCR (n = 11). (B) miR-186 level (percentage of HDMECs) in purified HDMECs that were cultured alone (HDMEC) or together with NCI-H460 cells (HDMEC (H460)), as assessed by qRT-PCR (n = 3). (C) miR-186 level (percentage of Con) in HDMECs that were treated for 24 h with vehicle (Con), 50 ng/mL VEGF, 100 ng/mL EGF, 50 ng/mL bFGF, 100 ng/mL IL-6, or 10 ng/mL TNF-α in EBM, as assessed by qRT-PCR (n = 3). (D) miR-186 level (percentage of normoxia) in HDMECs that were cultured for 24 h under normoxia or hypoxia, as assessed by qRT-PCR (n = 3). (E) Expression level of miR-186 or pri-miR-186 (percentage of Con) in HDMECs that were exposed for 24 h to vehicle (Con) or 100 μM CoCl_2_, as assessed by qRT-PCR (n = 3). (F) miR-186 level (percentage of Con) in HDMECs that were exposed for 24 h to vehicle (Con), 5 μM Bay 87-2243, or 5 μM Bay 11-7082, as assessed by qRT-PCR (n = 3). Means ± SEM. ∗p < 0.05, ∗∗p < 0.01, ∗∗∗p < 0.001 versus NEC, Con, or normoxia (A, paired Student’s t test; B, D, and E, unpaired Student’s t test; C and F, one-way ANOVA with Tukey’s multiple comparisons test).

ECs within the NSCLC tissues and matched adjacent non-tumor lung tissues of 11 human patients (Table S1) were excised by means of laser capture microdissection (LCM). These isolated ECs were then processed for real-time qRT-PCR analysis to assess miR-186 expression. By doing this, we found that miR-186 is significantly downregulated in tumor-associated ECs (TECs) of NSCLC patients compared with normal ECs (NECs) (Figure 1A).

### Regulation of miR-186 expression in ECs

Previous studies suggested that tumor cells are able to interact with ECs directly via gap junctions and adhesion receptors as well as indirectly through releasing soluble factors into the tumor microenvironment.^13^ To investigate whether the downregulation of miR-186 in TECs is induced by NSCLC cells, we cultured human dermal microvascular ECs (HDMECs) alone or co-cultured them with NSCLC NCI-H460 cells for 24 h. HDMECs were then isolated using CD31 MicroBeads, and their high purity in both the mono-culture group (>99%) and the co-culture group (>90%) was verified by flow cytometry. Further qRT-PCR assays showed that miR-186 is comparably expressed in HDMECs co-cultured with and without NCI-H460 cells (Figure 1B), indicating that neither the direct contact between tumor cells and ECs nor soluble factors secreted by the tumor cells causes the downregulation of endothelial miR-186. To further confirm this finding, we stimulated HDMECs with several pro-angiogenic factors that have been reported to be secreted by NSCLC cells, including vascular endothelial growth factor (VEGF), epidermal growth factor (EGF), basic fibroblast growth factor (bFGF), interleukin-6 (IL-6), and tumor necrosis factor alpha (TNF-α). ^14^^,^^15^^,^^16^^,^^17^ For this purpose, we used concentrations of the individual factors that have been previously described to stimulate angiogenesis. ^18^^,^^19^^,^^20^^,^^21^ As expected, none of these factors affected the expression of miR-186 (Figure 1C). In contrast, the expression of miR-186 was significantly suppressed in HDMECs that were exposed to hypoxia for 24 h compared with those cultured under normoxic conditions (Figure 1D).

Since HIF1α represents the most important transcriptional factor that mediates the pro-angiogenic response to hypoxia in tumors through regulating the transcription of a broad spectrum of genes and non-coding RNAs,^5^^,^^22^ we next investigated whether HIF1α inhibits the transcription of miR-186 in ECs. For this purpose, HDMECs were treated with the specific HIF1α inducer cobalt chloride (CoCl_2_) and the HIF1α inhibitor Bay 87-2243 for 24 h followed by qRT-PCR analysis. CoCl_2_ efficiently reduced the expression level of mature miR-186 in HDMECs by 56% (Figure 1E), whereas Bay 87-2243 significantly increased miR-186 expression by 26% (Figure 1F). Importantly, the intracellular level of primary miR-186 (pri-miR-186) was also significantly decreased by the treatment with CoCl_2_ (Figure 1E). In addition, we investigated whether miR-186 is also transcriptionally regulated by nuclear factor (NF)-κB, a transcription factor that is well-known to be activated by hypoxia.^23^ Our qRT-PCR data showed that inhibition of NF-κB by Bay 11-7082 does not affect miR-186 expression (Figure 1F). Noteworthy, 5 μM Bay 11-7082 was used in this assay. At this dosage, the compound is non-cytotoxic but effectively inhibits the activity of NF-κB in HDMECs, as demonstrated in a previous study.^24^

### Effects of miR-186 on the angiogenic activity of ECs

To investigate the function of miR-186 in regulating EC angiogenesis, HDMECs were transfected for 48 h with miR-186 mimic (miR-186m) or miR-186 inhibitor (miR-186i) to up- or downregulate intracellular miR-186, respectively. HDMECs that were transfected with a negative control of miRNA mimic (NCm) or a negative control of miRNA inhibitor (NCi) were assigned to the control groups. To verify the success of these interventions, the transfection efficiencies of miR-186m (5 and 10 nM) and miR-186i (50 and 100 nM) were analyzed by qRT-PCR assays, as shown in Figure S1. Based on these measurements, 5 nM miR-186m and 100 nM miR-186i were chosen for further *in vitro* assays.

**Figure 2:**
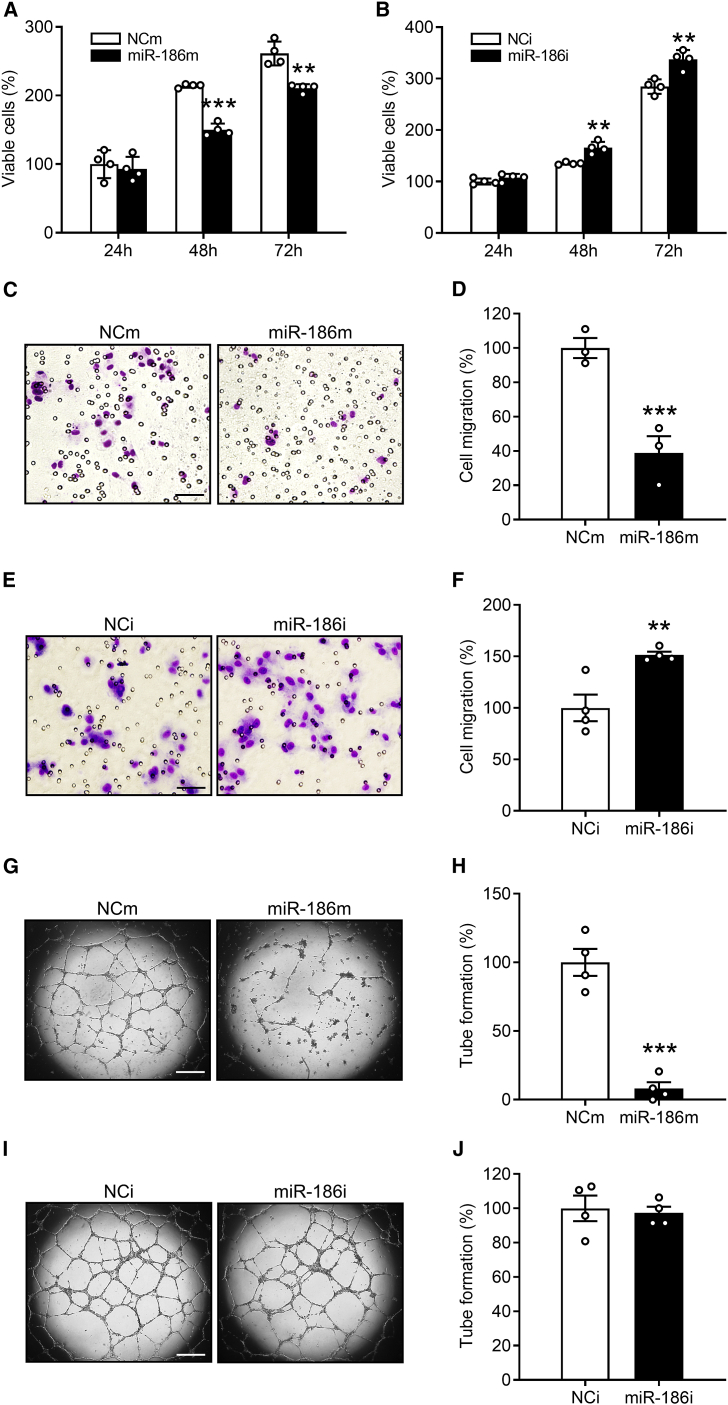
MiR-186 inhibits EC proliferation, migration, and tube formation (A and B) Number of viable HDMECs (percentage of NCm or NCi at 24 h), as assessed by WST-1 assay (n = 4). The cells transfected with NCm (A), miR-186m (A), NCi (B), or miR-186i (B) were seeded in 96-well plates and cultured for the indicated time periods. (C and E) Light microscopy images of migrated HDMECs that were transfected with NCm (C), miR-186m (C), NCi (E) or miR-186i (E). Scale bars, 70 μm (C) and 35 μm (E). (D and F) Migration (percentage of NCm or NCi) of HDMECs that were transfected with NCm (D), miR-186m (D), NCi (F), or miR-186i (F), as assessed by Transwell migration assay (n = 3–4). (G and I) Phase-contrast microscopy images of HDMEC-formed tubes. The cells were transfected with NCm (G), miR-186m (G), NCi (I), or miR-186i (I). Scale bar, 750 μm. (H and J) Tube formation (percentage of NCm or NCi) of HDMECs that were transfected with NCm (H), miR-186m (H), NCi (J), or miR-186i (J), as assessed by tube formation assay (n = 4). Means ± SEM. ∗∗p < 0.01, ∗∗∗p < 0.001 versus NCm or NCi (unpaired Student’s t test).

After the transfection period of 48 h, the HDMECs were trypsinized, collected, and counted. An identical number of transfected ECs in each group was then seeded into 96-well plates. Subsequently, the effects of miR-186 on EC viability and proliferation were assessed by means of a water-soluble tetrazolium (WST)-1 assay. Transfection of HDMECs with miR-186m significantly reduced the number of viable cells only at later time points (48 and 72 h of incubation) (Figure 2A). In contrast, miR-186i transfection significantly increased the number of viable HDMECs at these time points (Figure 2B). Next, we investigated the effects of miR-186 on EC migration by performing Transwell migration assays. Transfection with miR-186m efficiently reduced the number of migrated HDMECs by 85% (Figures 2C and 2D), whereas miR-186i significantly enhanced HDMEC migration by 51% (Figures 2E and 2F). Moreover, tube formation assays revealed that miR-186m strongly suppresses the tube-forming activity of HDMECs (Figures 2G and 2H), while miR-186i exerts no effects (Figures 2I and 2J).

**Figure 3:**
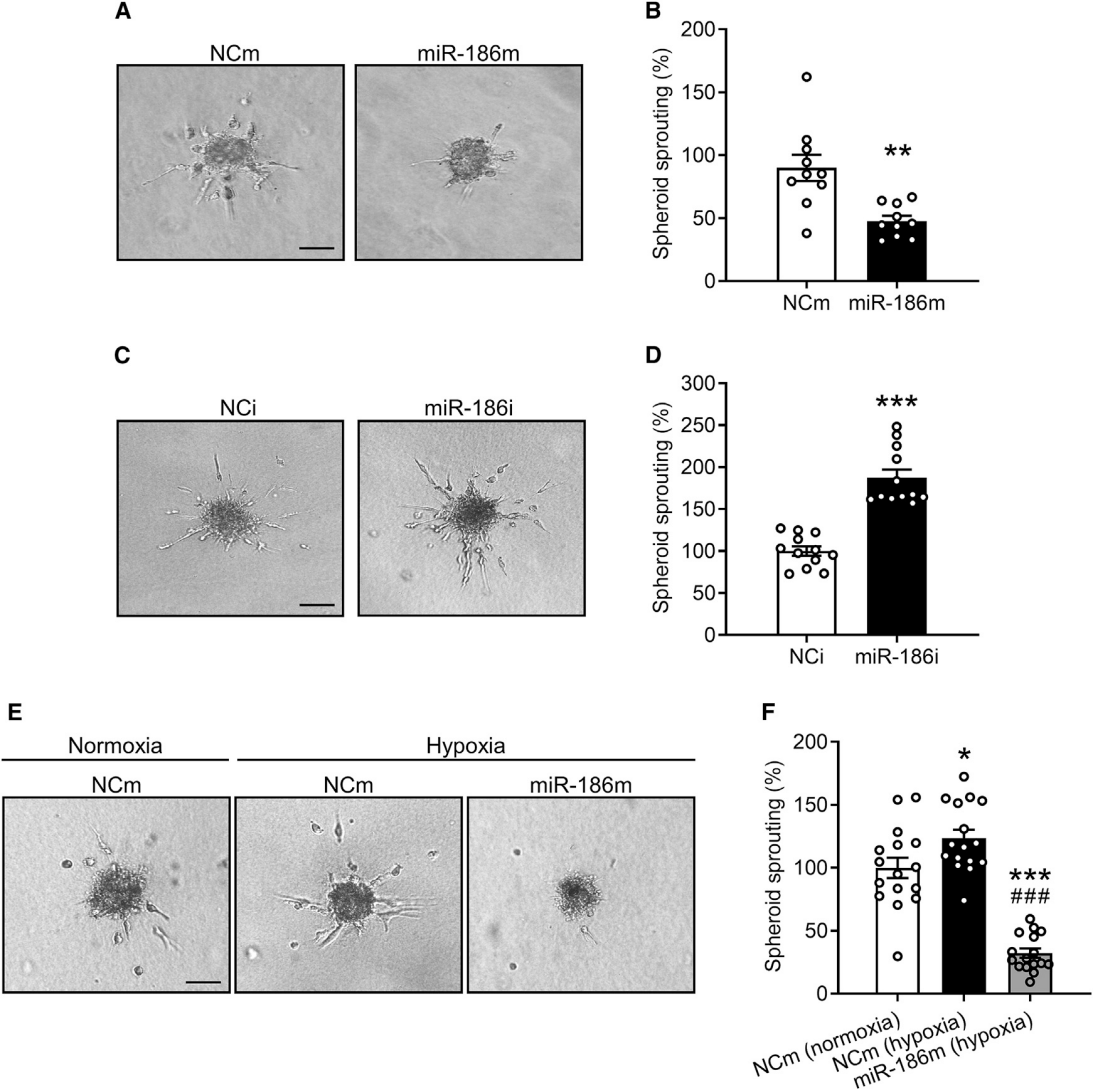
MiR-186 suppresses EC spheroid sprouting (A and C) Phase-contrast microscopy images of HDMEC spheroids. The HDMECs were transfected with NCm (A), miR-186m (A), NCi (C), or miR-186i (C). Scale bar, 85 μm. (B and D) Spheroid sprouting (percentage of NCm or NCi) of HDMECs that were transfected with NCm (B), miR-186m (B), NCi (D), or miR-186i (D), as assessed by spheroid sprouting assay (n = 12–14). (E) Phase-contrast microscopy images of HDMEC spheroids. The spheroids of NCm- or miR-186m-transfected HDMECs were incubated under normoxia or hypoxia for 24 h. Scale bar, 85 μm. (F) Spheroid sprouting (percentage of NCm [normoxia]) of HDMECs that were treated as described in (E), as assessed by spheroid sprouting assay (n = 16). Means ± SEM. ∗p < 0.05, ∗∗p < 0.01, ∗∗∗p < 0.001 versus NCm, NCi, or NCm (normoxia) (B and D, unpaired Student’s t test; F, one-way ANOVA with Tukey’s multiple comparisons test). ^###^p < 0.05 versus NCm (hypoxia) (F, one-way ANOVA with Tukey’s multiple comparisons test).

In addition, we performed three-dimensional HDMEC spheroid sprouting assays. After incubating HDMEC spheroids in a collagen matrix for 24 h, many ECs sprouted out from the spheroids of the NCm-transfected control group and invaded the surrounding matrix (Figures 3A and 3B). This process was markedly suppressed by transfecting HDMECs with miR-186m (Figures 3A and 3B). In contrast, miR-186i efficiently enhanced HDMEC spheroid sprouting (Figures 3C and 3D). Importantly, overexpression of miR-186 significantly reversed hypoxia-induced EC spheroid sprouting (Figures 3E and 3F), indicating that downregulation of endothelial miR-186 crucially contributes to hypoxia-triggered EC angiogenesis.

### Effects of endothelial miR-186 on *in vivo* angiogenesis and tumor growth

**Figure 4:**
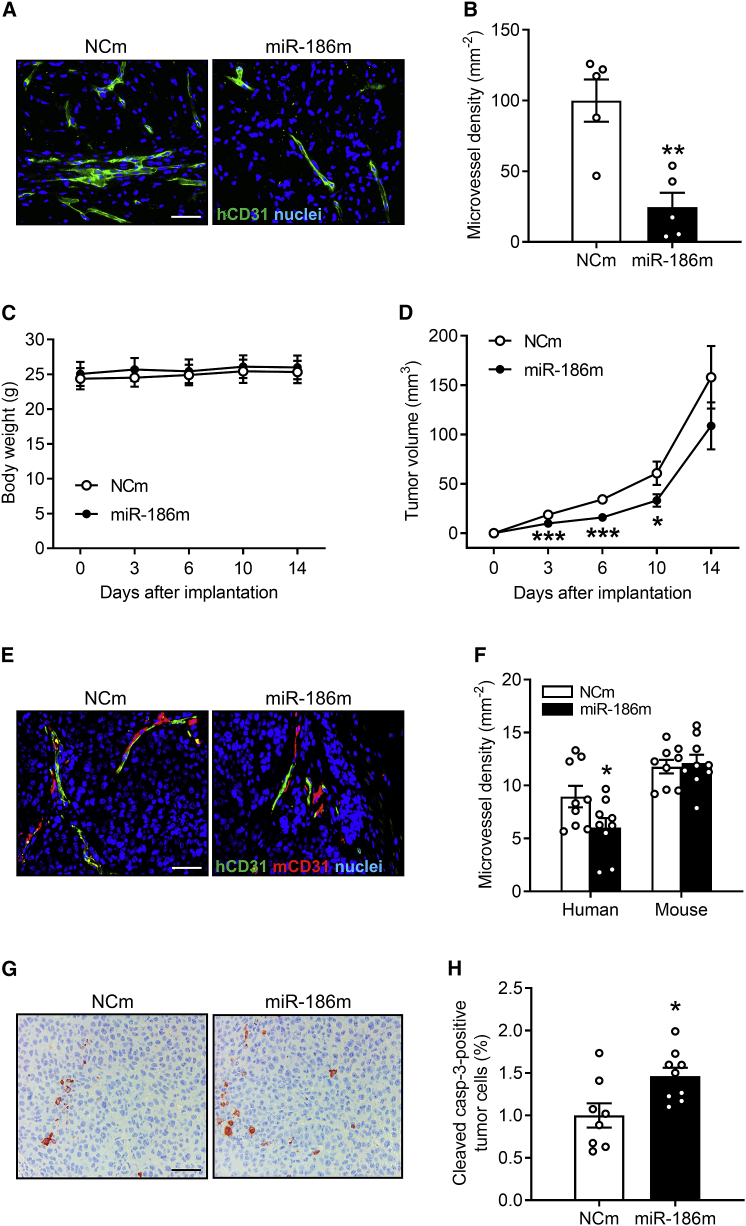
MiR-186 inhibits *in vivo* angiogenesis and tumor growth (A) Immunohistochemical staining of microvessels (green) in Matrigel plugs containing NCm- or miR-186m-transfected HDMECs. Cell nuclei (blue) are visualized by staining the sections with Hoechst 33342. Scale bar, 55 μm. (B) Microvessel density (mm^−2^) of Matrigel plugs containing NCm- or miR-186m-transfected HDMECs, as assessed by immunohistochemistry (n = 5). (C) Body weight (g) of NOD-SCID mice on day 0, 3, 7, 10, and 14 after implantation of tumors composed of NCI-H460 and NCm- or miR-186m-transfected HDMECs (n = 9). (D) Volume (mm³) of growing tumors composed of NCI-H460 and NCm- or miR-186m-transfected HDMECs, as assessed by caliper measurement (n = 9). (E) Immunohistochemical staining of human (green) and mouse (red) microvessels in tumors composed of NCI-H460 and NCm- or miR-186m-transfected HDMECs on day 14 after tumor implantation. Cell nuclei (blue) are visualized by staining the sections with Hoechst 33342. Scale bar, 55 μm. (F) Density (mm^−2^) of human and mouse microvessels in tumors composed of NCI-H460 and NCm- or miR-186m-transfected HDMECs on day 14 (n = 9), as assessed by immunohistochemistry. (G) Immunohistochemical staining of cleaved casp-3-positive tumor cells in xenografts composed of NCI-H460 and NCm- or miR-186m-transfected HDMECs. Scale bar, 55 μm. (H) Cleaved casp-3-positive tumor cells (percentage of the total tumor cells) in xenografts composed of NCI-H460 and NCm- or miR-186m-transfected HDMECs (n = 8–9), as assessed by immunohistochemistry. Means ± SEM. ∗p < 0.05, ∗∗p < 0.01, ∗∗∗p < 0.001 versus NCm (unpaired Student’s t test).

To confirm the inhibitory effects of miR-186 on angiogenesis *in vivo*, we first performed a Matrigel plug assay. For this assay, a cell mixture composed of NCm- or miR-186m-transfected HDMECs and Matrigel were injected into immunodeficient CD1 nude mice. On day 7 after implantation, the Matrigel plugs containing miR-186m-transfected HDMECs exhibited a significantly lower microvessel density compared with those of the NCm-transfected control group (Figures 4A and 4B).

We further investigated whether endothelial miR-186 inhibits tumor angiogenesis and growth. For this purpose, a flank tumor model was performed by injecting NOD-SCID mice with NCI-H460 cells admixed with NCm- or miR-186m-transfected HDMECs into the flanks. Noteworthy, the induced tumors did not affect the body weight (Figure 4C) and general behavior of the mice. By analyzing the volume of the developing tumors, we could demonstrate that transfection of HDMECs with miR-186m inhibits NCI-H460 tumor growth on days 3, 7, and 10 (Figure 4D). As expected, miR-186 overexpression in HDMECs significantly inhibited the development of human microvessels within the tumors but did not affect the ingrowth of mouse microvessels (Figures 4E and 4F). Additional immunohistochemical stainings revealed that NCI-H460 xenografts containing miR-186m-transfected HDMECs possess a higher fraction of cleaved caspase (casp)-3-positive apoptotic tumor cells (Figures 4G and 4H).

### Validation of protein kinase C alpha as a target of miR-186

To identify the molecular targets mediating the anti-angiogenic function of miR-186, we first analyzed the validated human targets of this miRNA based on the current literature. We found 12 genes that are associated with angiogenesis (Figure S2A), including mitogen-activated protein kinase kinase kinase 4 (MAP3K4), transforming growth factor β receptor 2 (TGFBR2), hypoxia-inducible factor 1 alpha (HIF1A), MAP3K2, RELA (p65), Jagged1 (JAG1), high-mobility group box 1 (HMGB1), Rho-associated coiled-coil containing protein kinase 1 (ROCK1), yes-associated protein 1 (YAP1), SMAD family member 6 (SMAD6), cell division protein kinase 6 (CDK6), and cell division cycle 42 (CDC42). However, among these genes, only the mRNA levels of YAP1 and CDC42 were slightly reduced in miR-186m-transfected HDMECs (Figure S2B). These findings indicate that these 12 genes may not be relevant functional targets of miR-186 and therefore they were not evaluated further.

**Figure 5:**
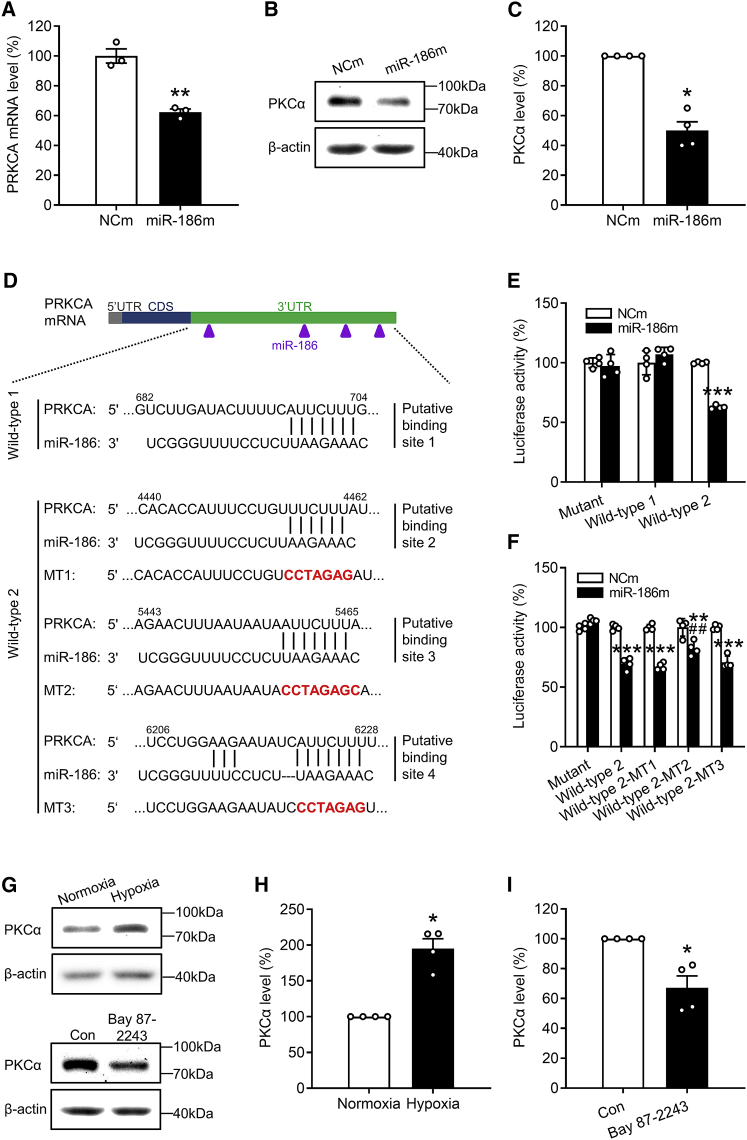
MiR-186 targets PRKCA in ECs (A) mRNA level (percentage of NCm) of PRKCA in HDMECs that were transfected with NCm or miR-186, as assessed by qRT-PCR (n = 3). (B) Representative western blots of PKCα and β-actin expression in NCm- or miR-186-transfected HDMECs. (C) Expression level (percentage of NCm) of PKCα in HDMECs that were transfected with NCm or miR-186, as assessed by western blotting (n = 4 independent experiments). (D) The four putative miR-186 binding sites within the 3′ UTR of human PRKCA mRNA. CDS, coding sequence. (E) Luciferase activity (percentage of NCm-transfected mutant group) in HEK293T cells that were co-transfected with NCm or miR-186m, Renilla luciferase plasmid, and firefly luciferase plasmid without (mutant) or with wild-type sequences of PRKCA-3′ UTR, which contained putative binding site 1 (wild-type 1) or putative binding sites 2–4 (wild-type 2), as assessed by dual-luciferase assay (n = 4). (F) Luciferase activity (percentage of NCm-transfected mutant group) in HEK293T cells that were co-transfected with NCm or miR-186m, Renilla luciferase plasmid, and firefly luciferase plasmid without (mutant) or with wild-type 2 sequences of PRKCA-3′ UTR, which contained mutation in the individual putative binding site 2–4 or not, as assessed by dual-luciferase assay (n = 4). (G) Representative western blots of PKCα and β-actin expression in HDMECs that were incubated under normoxia or hypoxia for 72 h (upper) or in HDMECs that were exposed to vehicle (Con) or 5 μM Bay 87-2243 for 72 h (lower). (H and I) Expression level (percentage of normoxia or Con) of PKCα in HDMECs that were incubated under normoxia or hypoxia (H) or in HDMECs that were exposed to vehicle (Con) or Bay 87-2243 (I), as assessed by western blotting (n = 4 independent experiments). Means ± SEM. ∗p < 0.05, ∗∗p < 0.01, ∗∗∗p < 0.001 versus NCm, normoxia, or Con (A, E, and F, unpaired Student’s t test; C, H, and I, Mann-Whitney U test). ^##^p < 0.05 versus miR-186m-transfected wild-type 2 group (F, unpaired Student’s t test).

Next, we analyzed the predicted human target genes of miR-186 according to the algorithm of miRDB and TargetScan and detected six angiogenesis-associated genes (Figure S2C). These genes encode frizzled class receptor 3 (FZD3), metadherin (MTDH), ribosomal protein S6 kinase A3 (RSP6KA3), SATB homeobox 1 (SATB1), sirtuin 1 (SIRT1), and protein kinase C alpha (PKCα). By means of qRT-PCR, the mRNA level of protein kinase C alpha gene (PRKCA) was found to be significantly downregulated in HDMECs transfected with miR-186m (Figures 5A and S2D). Consistently, the protein level of PKCα was also markedly decreased in miR-186m-overexpressing HDMECs (Figures 5B and 5C).

As predicted by TargetScan, there are four putative binding sites of miR-186 in the 3′ UTR of PRKCA mRNA, but no putative binding sites in the mRNA of the other PRKC family members, including PRKCB, PRKCD, PRKCE, PRKCG, PRKCH, PRKCI, PRKCQ, and PRKCZ (Figure S3). To verify whether PRKCA is a direct target of miR-186, we constructed two PRKCA-3′ UTR firefly luciferase plasmids that contained a different part of the 3′ UTR sequence of PRKCA. One was named wild-type 1 and contained one putative binding site of miR-186. The other one, containing three putative binding sites, was named wild-type 2 (Figure 5D). Then, we co-transfected miR-186m, Renilla luciferase plasmid, and plasmid wild-type 1, wild-type 2, or an empty plasmid without PRKCA-3′ UTR (mutant) into HEK293T cells followed by a dual-luciferase assay. MiR-186m significantly diminished the luciferase activity of the wild-type 2 plasmid, but not that of the wild-type 1 or mutant plasmid (Figure 5E). These results demonstrate that miR-186 preferentially binds to the 3′ UTR sequence of PRKCA in the wild-type 2 plasmid. To clarify which one of the three putative binding sites was responsible for the observed miR-186-induced PRKCA downregulation, a mutation of each putative binding site was introduced into the wild-type 2 plasmid, named wild-type 2-MT1, wild-type 2-MT2, and wild-type 2-MT3, respectively (Figure 5D). By means of further dual-luciferase assays, we observed that the mutation in the putative binding site 3 (MT2), but not in the binding site 2 (MT1) or 4 (MT3), significantly counteracts the miR-186-induced reduction of luciferase activity of the wild-type 2 plasmid (Figure 5F).

Because hypoxia downregulates endothelial miR-186 by activating HIF1α, we next analyzed the expression of PKCα in HDMECs that were incubated for 72 h under normoxic or hypoxic conditions. Western blot analyses showed that hypoxia significantly increases the protein level of PKCα in ECs by 95% compared with normoxia (Figures 5G and 5H). In contrast, inhibition of HIF1α with Bay 87-2243 repressed the expression of PKCα by 30% (Figures 5G–5I). These results further confirmed PRKCA as a target of miR-186.

### Involvement of PRKCA in miR-186 function

**Figure 6:**
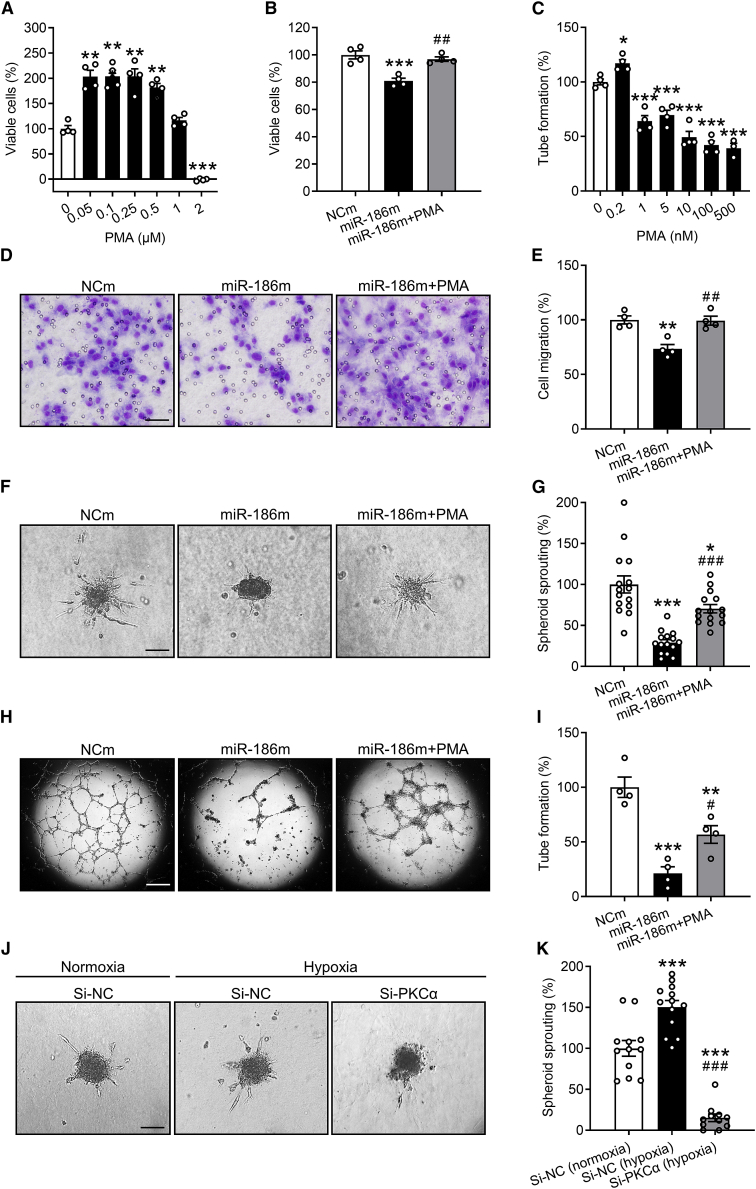
MiR-186 inhibits angiogenesis by targeting PRKCA (A) Number of viable HDMECs (percentage of 0 μM), as assessed by WST-1 assay (n = 4). The cells were exposed for 72 h to a serial dilution of PMA. (B) Number of viable HDMECs (percentage of NCm), as assessed by WST-1 assay (n = 4). The cells were transfected with NCm or miR-186m and then treated with or without 0.5 μM PMA for 72 h. (C) Tube formation (percentage of 0 nM) of HDMECs that were exposed for 24 h to different concentrations of PMA, as assessed by tube formation assay (n = 4). (D) Light microscopy images of migrated HDMECs that were transfected with NCm or miR-186m and then treated with or without 0.5 μM PMA. Scale bar, 35 μm. (E) Migration (percentage of NCm) of HDMECs that were treated as described in (D), as assessed by Transwell migration assay (n = 4). (F) Phase-contrast microscopy images of HDMEC spheroids. The spheroids of NCm- or miR-186m-transfected HDMECs were treated with or without 0.5 μM PMA for 24 h. Scale bar, 85 μm. (G) Spheroid sprouting (percentage of NCm) of HDMECs that were treated as described in (F), as assessed by spheroid sprouting assay (n = 15). (H) Phase-contrast microscopy images of HDMEC-formed tubes. The cells were transfected with NCm or miR-186m and then treated with or without 0.2 nM PMA for 24 h. Scale bar, 750 μm. (I) Tube formation (percentage of NCm) of HDMECs that were treated as described in (H), as assessed by tube formation assay (n = 4). (J) Phase-contrast microscopy images of HDMEC spheroids. The spheroids of si-NC- or si-PKCα-transfected HDMECs were incubated under normoxia or hypoxia for 24 h. Scale bar, 85 μm. (K) Spheroid sprouting (percentage of si-NC [normoxia]) of HDMECs that were treated as described in (J), as assessed by spheroid sprouting assay (n = 11–13). Means ± SEM. ∗p < 0.05, ∗∗p < 0.01, ∗∗∗p < 0.001 versus 0 μM, NCm, 0 nM, or si-NC (normoxia) (one-way ANOVA with Tukey’s multiple comparisons test). ^#^p < 0.05, ^##^p < 0.01, ^###^p < 0.001 versus miR-186m or si-NC (hypoxia) (B, E, G, I, and K, one-way ANOVA with Tukey’s multiple comparisons test).

PKCα has been reported to play an important role in angiogenesis.^25^^,^^26^^,^^27^ To determine whether miR-186 inhibits angiogenesis through targeting PRKCA, a well-established PKCα activator phorbol 12-myristate 13-acetate (PMA) was used for rescue experiments. For this, we first performed a WST-1 assay showing that 0.05–0.5 μM PMA significantly increases the number of viable HDMECs (Figure 6A). Accordingly, 0.5 μM PMA was used for the following WST-1, migration, and spheroid sprouting assays. These assays revealed that treatment with PMA largely reverses the miR-186m-induced reduction of HDMEC proliferation, migration, and spheroid sprouting (Figures 6B and 6D–6G). Of note, in tube formation assays, we treated miR-186m-transfected HDMECs with 0.2 nM PMA, instead of 0.5 μM, because PMA at a dosage higher than 0.2 nM could not promote but inhibited HDMEC tube formation (Figure 6C). In doing so, we demonstrated that activation of PKCα by PMA also significantly reverses the inhibitory effect of miR-186m on EC tube formation (Figures 6H and 6I). These findings were confirmed by two additional PKCα stimulators, bryostatin 1 and phorbol 12, 13-dibutyrate (PDBu). Based on the results of WST-1 assays, 100 nM bryostatin 1 and 1 μM PDBu were chosen for spheroid sprouting assays (Figures S4A and S4B). Our results showed that, similar to PMA, bryostatin 1 and PDBu significantly counteract miR-186m-suppressed HDMEC spheroid sprouting (Figures S4C and S4D).

Moreover, to determine the involvement of PKCα in hypoxia-induced angiogenesis, we downregulated the intracellular level of PKCα by transfecting HDMECs with its small interfering RNAs (si-PKCα). Cells transfected with negative control of siRNA (si-NC) served as controls. The transfection efficiency of si-PKCα was assessed by western blotting (Figure S5). Knockdown of PKCα markedly inhibited the hypoxia-induced spheroid sprouting of HDMECs (Figures 6J and 6K). This indicates that upregulation of PKCα essentially contributes to hypoxia-stimulated EC angiogenic activity.

### Action of extracellular signal-regulated kinase as a key downstream factor of the miR-186-PRKCA axis

**Figure 7:**
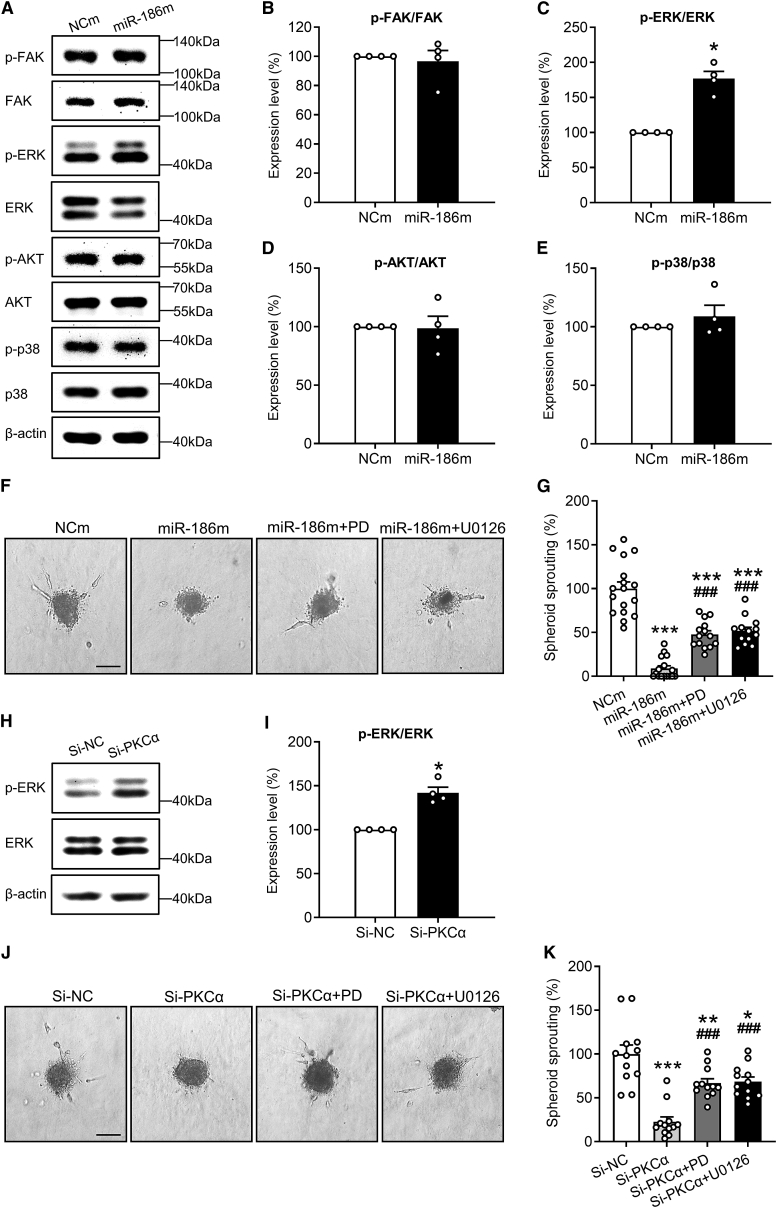
ERK phosphorylation mediates the anti-angiogenic effect of the miR-186-PRKCA axis (A) Representative western blots of p-FAK, FAK, p-ERK, ERK, p-AKT, AKT, p-p38, p38, and β-actin expression in NCm- or miR-186-transfected HDMECs. (B–E) Expression level (percentage of NCm) of p-FAK/FAK (B), p-ERK/ERK (C), p-AKT/AKT (D), and p-p38/p38 (E) in HDMECs that were transfected with NCm or miR-186, as assessed by western blotting (n = 4 independent experiments). (F) Phase-contrast microscopy images of HDMEC spheroids. The spheroids of NCm- or miR-186m-transfected HDMECs were treated with or without the MEK inhibitor PD0325901 (PD) and U0126 for 24 h. Scale bar, 85 μm. (G) Spheroid sprouting (percentage of NCm) of HDMECs that were treated as described in (F), as assessed by spheroid sprouting assay (n = 13–17). (H) Representative western blots of p-ERK, ERK, and β-actin expression in si-NC- or si-PKCα-transfected HDMECs. (I) Expression level (percentage of si-NC) of p-ERK/ERK in HDMECs that were transfected with si-NC or si-PKCα, as assessed by western blotting (n = 4 independent experiments). (J) Phase-contrast microscopy images of HDMEC spheroids. The spheroids of si-NC- or si-PKCα-transfected HDMECs were treated with or without PD or U0126 for 24 h. Scale bar, 85 μm. (K) Spheroid sprouting (percentage of si-NC) of HDMECs that were treated as described in (J), as assessed by spheroid sprouting assay (n = 12). Means ± SEM. ∗p < 0.05, ∗∗p < 0.01, ∗∗∗p < 0.001 versus NCm or si-NC (B, C, D, E, and I, Mann-Whitney U test; G and K, one-way ANOVA with Tukey’s multiple comparisons test). ^###^p < 0.001 versus miR-186m or si-PKCα (G and K, one-way ANOVA with Tukey’s multiple comparisons test).

To identify the downstream pathways that mediate the anti-angiogenic function of the miR-186-PRKCA axis, we first investigated the effects of miR-186m on several important angiogenesis-associated signaling pathways, including focal adhesion kinase (FAK), AKT, p38, and extracellular signal-regulated kinase (ERK) pathways. ^4^^,^^28^^,^^29^^,^^30^^,^^31^ Western blot analyses revealed that miR-186m significantly increases the level of phosphorylated ERK (p-ERK) but induces no change in the phosphorylation level of FAK, AKT, and p38 (Figures 7A–7E). Of interest, inhibition of ERK phosphorylation with the mitogen-activated protein kinase/ERK kinase (MEK) inhibitors PD0325901 and U0126 significantly counteracted miR-186m-suppressed HDMEC spheroid sprouting (Figures 7F and 7G). Moreover, knockout of PKCα with si-PKCα also promoted ERK phosphorylation (Figures 7H and 7I), while PD0325901 and U0126 largely reversed si-PKCα-reduced EC spheroid sprouting (Figures 7J and 7K). These findings indicate that miR-186 inhibits angiogenesis through directly targeting PRKCA and consequently phosphorylating ERK in ECs.

## Discussion

MiR-186 is expressed in various types of human cancer cells, and its functions in regulating tumor cell activities have been extensively studied. ^10^ For instance, miR-186 has been shown to inhibit NSCLC cell proliferation, migration, invasion, and drug resistance.^32^^,^^33^^,^^34^^,^^35^ Moreover, the tube-forming activity of retinoblastoma was suppressed by miR-186, indicating an inhibitory effect of this miRNA on tumor cell vasculogenic mimicry.^36^ Nevertheless, the effect of miR-186 on EC activity during tumor angiogenesis still needs to be elucidated. In the present study, we close this gap of knowledge by demonstrating that miR-186 efficiently inhibits the angiogenic activity of ECs through targeting PRKCA. Under hypoxic conditions, which typically occur in the tumor microenvironment, the expression of this miRNA in ECs is downregulated by transcription factor HIF1α. Taken together, these findings indicate that endothelial miR-186 is an important inhibitor of tumor angiogenesis and, thus, represents a promising target for anti-angiogenic treatment strategies.

According to GLOBOCAN 2020 estimates of cancer incidence and mortality, lung cancer is the second commonly diagnosed cancer worldwide and remains the leading cause of cancer death.^37^ NSCLC is responsible for 85% of lung cancer cases.^38^ Therefore, we concentrated on this specific cancer type and first analyzed miR-186 expression in ECs dissected from human NSCLC tissues and matched non-cancer lung tissues. Of interest, miR-186 was significantly downregulated in TECs. However, additional *in vitro* analyses showed that co-culturing HDMECs with human NSCLC NCI-H460 cells does not affect the miR-186 level in HDMECs. In line with this result, a previous study reported a constant expression of miR-186 in human brain microvascular ECs that were cultured in the presence or absence of human glioblastoma U87 cells.^39^ These findings indicate that the downregulation of endothelial miR-186 is not induced by tumor cells. This conclusion is further supported by our observation that stimulation of HDMECs with the pro-angiogenic factors VEGF, EGF, bFGF, IL-6, and TNF-α, which are known to be secreted by tumor cells,^14^^,^^15^^,^^16^^,^^17^ has no influence on the expression of endothelial miR-186.

Hypoxia is a key microenvironmental factor in tumors that stimulates the angiogenic activity of ECs.^5^ Therefore, we incubated HDMECs under hypoxic or normoxic conditions. Of interest, we observed a strong decrease in the miR-186 levels of hypoxia-exposed ECs. This is in line with a recent study showing that miR-186 expression in human umbilical vein ECs is reduced under hypoxia.^40^ To clarify the underlying mechanism, we investigated whether the two key downstream transcription factors of hypoxia, i.e., NF-κB and HIF1α, mediate hypoxia-reduced miR-186 expression. Zhao et al. reported that cytokine-activated NF-κB is able to bind to the promoter of miR-186 and to induce its expression in non-transformed cells. ^41^ However, we found that NF-κB is not involved in the regulation of endothelial miR-186 expression in our experimental setting. In contrast, HIF1α mediated the hypoxia-induced downregulation of miR-186 expression in ECs. Vice versa, Lin et al. reported that HIF1α can also be regulated by miR-186. In fact, they found that this miRNA suppresses cell proliferation through targeting HIF1α in the gastric cancer cell lines MKN45 and SGC7901.^42^ This indicates the complexity of the mutual regulation of miR-186 and HIF1α under different conditions and in different cell types.

We then investigated the function of miR-186 in angiogenesis. For this, HDMECs instead of TECs were used. This is because even freshly isolated TECs from tumor tissues are no longer real TECs, since they have already been out of the tumor microenvironment. In contrast, HDMECs are not only easily accessible but also possess potent angiogenic activity under normal culture conditions. They are therefore widely used by many researchers for *in vitro* angiogenesis assays.^43^ In the present study, these assays demonstrated a pleiotropic anti-angiogenic activity of miR-186. In fact, miR-186 targeted all the key angiogenic activities of ECs, including their proliferation, migration, tube formation, and spheroid sprouting. Of note, these effects were not caused by the cytotoxicity of miR-186. This view is supported by the fact that the angiogenesis assays were performed with NCm- and miR-186m-transfected ECs that did not differ in their viability during the first 24-h incubation after their transfection. To confirm the *in vitro* findings, we additionally carried out a Matrigel plug assay, which is a reliable and widely used *in vivo* assay for the evaluation of pro- and anti-angiogenic factors.^44^ We demonstrated that miR-186 overexpression in HDMECs significantly inhibits the vascularization of Matrigel plugs. For this assay, we analyzed the density of human CD31-positive microvessels by means of immunohistochemistry. This parameter is more direct and accurate than the plasma volume or the total amount of hemoglobin within the plugs, since the latter two parameters are largely dependent on the diameter of microvessels and hemorrhages within the implants as well as the plug size.^45^

In an additional *in vivo* approach, we injected immunodeficient mice with a mixture of NCI-H460 cells and HDMECs. We observed that overexpression of miR-186 in HDMECs significantly suppresses their assembly into new microvessels within the developing tumors, resulting in a reduced tumor volume on days 3, 7, and 10 compared with controls. However, the size of tumors containing either NCm- or miR-186-transfected HDMECs did not differ anymore on day 14. This may be explained by the fact that the intracellular level of transfected miR-186m was diluted over time due to the division of HDMECs. Moreover, it should be considered that the present flank tumor model permits miR-186 overexpression in exogenous human ECs but not endogenous mouse ECs, which migrate into the developing tumors from the surrounding host tissue throughout the time course of the experiments. Therefore, the inhibitory effect of miR-186 on NSCLC angiogenesis and growth may be largely underestimated in this model.

To unravel the molecular mechanisms of miR-186 function, we first assessed the mRNA levels of 12 validated human targets of this miRNA that are known to be involved in angiogenesis. Unexpectedly, only YAP1 and CDC42 mRNA were slightly reduced in miR-186m-transfected HDMECs. These results strongly support the view that miRNA functions differently in diverse cell types.^46^ Therefore, it is necessary to identify the specific targets of miR-186 in ECs. To achieve this, we further analyzed the expression of six angiogenesis-related putative human targets of miR-186 predicted by the miRDB and TargetScan databases. We found that PRKCA mRNA is markedly downregulated by miR-186. By means of dual-luciferase assays, we could demonstrate that PRKCA is a direct target of miR-186. Although there are four putative binding sites of miR-186 in the 3′ UTR of the human PRKCA mRNA sequence, only one of them (at position 5,458–5,465 of PRKCA-3′ UTR) mediated the miR-186-induced PRKCA mRNA repression. The other three binding sites probably had a lower miR-186-binding ability impeding the miRNA-mRNA duplex formation.

PKCα is a member of the PKC family of serine/threonine kinases that are activated by phosphatidylserine, diacylglycerol, and phorbol esters. ^47^ Its overexpression or activation has been shown to promote EC migration and tube formation.^25^^,^^26^ In contrast, inhibition of PKCα by its antisense oligonucleotides, si-PKCα, or the inhibitor Gö 6976 suppresses EC migration and tube formation *in vitro* as well as myocardial neovascularization *in vivo*.^26^^,^^27^ Accordingly, PKCα represents a potential target for anti-angiogenic therapy. To investigate whether downregulation of PKCα mediates the anti-angiogenic function of miR-186, we treated miR-186m-transfected HDMECs with the PKCα activators PMA, bryostatin 1, and PDBu. These compounds were used instead of a plasmid overexpressing PKCα, because, in preliminary experiments, the co-transfection efficiency of miR-186m and an overexpression vector was very low in HDMECs. We found that activation of PKCα significantly counteracts the miR-186m-induced reduction of EC angiogenic activity. This indicates that PRKCA is a functional target of miR-186 in ECs.

In conclusion, the present study demonstrates that hypoxia-induced downregulation of miR-186 in ECs promotes NSCLC angiogenesis by upregulating PKCα. Hence, endothelial miR-186 represents a promising target for the development of miRNA-based anti-angiogenic tumor therapies. A major prerequisite to achieve this is the development of miRNA modifications and sophisticated delivery systems, which enable a high efficiency and specificity of miRNA-based therapeutics.^48^^,^^49^ Moreover, it has to be guaranteed that such therapeutics are safe and do not induce severe side effects in patients.^50^^,^^51^ Tremendous progress in miRNA bioengineering, nanotechnology, and viral vector development may markedly contribute to fulfilling these criteria in future clinical practice.

## Materials and methods

### Study design

The sample size of each assay was determined according to previous publications. For *in vitro* assays, at least three independent experiments were conducted. Each experiment consisted of a minimum of three biological replicates, which were defined as independent cell cultures processed under the same condition. For mouse models, at least five animals were included in each group. No randomization was performed for the *in vivo* experiments. However, their data were collected and analyzed by investigators that were unaware of group assignment. All data were included for statistical analyses and no outliers were removed. The detailed n values for each assay are shown in the figure legends.

### Chemicals

The HIF1α activator CoCl_2_ and the NF-κB inhibitor Bay 11-7082 were purchased from Santa Cruz Biotechnology (Heidelberg, Germany). The MEK inhibitors PD0325901 and U0126 as well as the HIF1α inhibitor Bay 87-2243 were purchased from SelleckChem (Munich, Germany). The PKCα activators PMA, bryostatin 1, and PDBu were purchased from Tocris Biosciences (Bristol, UK).

### Cell culture

HDMECs were purchased from PromoCell (Heidelberg, Germany) and maintained in endothelial cell growth medium (ECGM)-MV (PromoCell). The human NSCLC cell line NCI-H460 was purchased from ATCC (Wesel, Germany) and incubated in RPMI 1640 medium supplemented with 10% fetal calf serum (FCS), 100 U/mL penicillin, and 0.1 mg/mL streptomycin (PAA, Cölbe, Germany). HEK293T cells were purchased from ATCC and cultured in DMEM (PAA) supplemented with 10% FCS, 100 U/mL penicillin, and 0.1 mg/mL streptomycin. These cells were cultured at 37°C in a humidified incubator with 5% CO_2_, 20% O_2_, and 75% N_2_. In a subset of experiments, hypoxia was achieved by incubating HDMECs or HDMEC spheroids in a hypoxia chamber (CB 53; Binder, Tuttlingen, Germany) with 5% CO_2_, 1% O_2_, and 94% N_2_ at 37°C for 24 or 72 h.

### Cell transfection

ECs were transfected for 48 h with miR-186m (Qiagen, Hilden, Germany) or miR-186i (Qiagen) using the HiPerFect reagent (Qiagen) to overexpress or knock down this miRNA. For the control groups, cells were transfected with NCm (Qiagen) or NCi (Qiagen). To downregulate the expression level of PKCα, HDMECs were transfected for 48 h with 120 nM si-PKCα (ON-TARGETplus siRNA SMARTpool, Dharmacon, Colorado, USA) using the HiPerFect reagent (Qiagen). Cells transfected with si-NC (Qiagen) served as controls. After transfection, ECs were trypsinized, collected, and counted. An identical number of transfected ECs in each group was then used for different angiogenesis assays.

### Co-culture of ECs with NSCLC cells

To investigate whether NSCLC cells affect the expression of miR-186 in HDMECs, contact co-culture experiments were carried out. For this purpose, 1 × 10^6^ HDMECs alone or together with 5 × 10^6^ NCI-H460 cells were cultured in 100-mm dishes with endothelial cell basal medium (EBM; PromoCell). After 24 h, ECs were purified using the human CD31 MicroBead kit (Miltenyi Biotec, Bergisch Gladbach, Germany). Briefly, the cells were detached, pelleted by centrifugation, and resuspended in EBM followed by addition of FcR blocking reagent and CD31 MicroBeads. After incubation at 4°C for 15 min, the cells were pelleted, resuspended in EBM and loaded onto an LS Column, which was placed in a magnetic MidiMACS separator. After washing with EBM three times, the column carrying ECs was removed from the separator. Then, ECs were flushed out of the column with EBM three times and processed for purity analysis and RNA extraction.

### Human patient samples

Eleven matched pairs of human NSCLC and non-cancerous adjacent lung tissues were obtained from Saarland University Hospital (Homburg, Saarland, Germany). The clinic-pathological characteristics of the included patients are described in Table S1. The analysis using human tissues was approved by the local ethics committee (permit number: 01/08) and the informed consent was obtained from all the included patients.

### LCM

To examine the expression of miR-186 in ECs within tumor and non-tumor lung tissues, ECs were exclusively isolated using the LCM technique. For this purpose, formalin-fixed paraffin-embedded (FFPE) tissue sections at 5 μm were cut and mounted on membrane slides (Leica Microsystems, Wetzlar, Germany). After staining the sections with hematoxylin and eosin, around 2,000 ECs were collected from each sample by using an LCM microscope (Leica AS LMD; Leica Microsystems) for RNA isolation.

### Flow cytometry

To assess EC purity after isolation, as previously described,^52^ the cells were stained with a mouse anti-human CD31 antibody conjugated to fluorescein isothiocyanate (1:100; BD Pharmingen; BD Biosciences, Heidelberg, Germany), followed by measurement with a FACScan flow cytometer (BD Biosciences). For each sample, more than 10,000 events were acquired and analyzed using the CellQuest Pro software (BD Biosciences).

### WST-1 assay

In this assay, the tetrazolium salt WST-1 is cleaved to soluble formazan by the cellular mitochondrial dehydrogenase. Therefore, the amount of generated formazan positively correlates with the number of viable cells. Briefly, HDMECs were seeded into each well of 96-well plates. After culture for the indicated periods of time, WST-1 reagent (Roche Diagnostics, Mannheim, Germany) was added to each well, followed by absorbance measurements in a microplate photometer (PHOmo; anthos Mikrosysteme, Krefeld, Germany).

### Migration assay

To assess EC migration, Transwell migration assays were carried out. Briefly, HDMECs were suspended in EBM and seeded into inserts with 8-μm pores in 24-Transwell plates (Corning; Merck, Darmstadt, Germany). EBM supplemented with 1% FCS was added to each well of plates beneath the insert. After 5 h of incubation, the non-migrated cells were removed and the migrated ones were dyed with Diff-Quick (LT-SYS, Berlin, Germany). The migrated cells were then photographed using a BZ-8000K microscope (Keyence, Osaka, Japan) and counted in 20 fields of each insert membrane at 200× magnification.

### Tube formation assay

The tube-forming activity of ECs was evaluated by a tube formation assay. For this purpose, 1.7 × 10^4^ HDMECs were seeded into each well of 96-well plates, which were coated with Matrigel (Corning). After 18 h of incubation, the EC-formed tubes were photographed by a phase-contrast microscope (BZ-8000; Keyence) and the number of tube meshes was analyzed using the ImageJ angiogenesis analyzer plugin (NIH, Bethesda, MD, USA).

### Spheroid sprouting assay

As previously described with minor modifications,^24^ HDMECs in ECGM-MV containing 20% methylcellulose (Thermo Fisher Scientific, Karlsruhe, Germany) were seeded into non-adherent 96-well round-bottom plates (500 cells per well). After culture for 24 h, the generated spheroids were suspended in a collagen solution. Then, approximately 50 spheroids were seeded into each well of 24-well plates. After incubation for 45 min, ECGM-MV supplemented with 3 ng/mL VEGF (R&D Systems, Abingdon, UK) was added into each well. After 24 h of incubation, the spheroids were photographed under a phase-contrast microscope (DFC450C; Leica Microsystems) and the cumulative sprouting length of each spheroid was quantified using the LAS V4.8 software (Leica Microsystems).

### Mouse models

All mouse experiments were performed in accordance with the German legislation for animal welfare and the Guide for the Care and Use of Laboratory Animals (Eighth Edition, 2011) and their protocols were approved by the local animal protection committee (permit number: 01/2019).

To evaluate the effects of miR-186 on angiogenesis *in vivo*, a Matrigel plug assay was conducted as previously described with some modifications.^53^ Briefly, NCm- or miR-186m-transfected HDMECs were suspended in ECGM-MV at 1 × 10^7^ cells/mL and mixed with growth factor-reduced Matrigel (high concentration; Corning) at a volume ratio of 1:1. Afterward, 300 μL of the HDMECs-Matrigel mixtures containing 1 μg/mL VEGF (R&D Systems), 1 μg/mL bFGF (R&D Systems), and 60 IU/mL heparin (Braun, Melsungen, Germany) were injected subcutaneously in the right and left abdominal wall of five immunodeficient male CD1 nude mice (age, 8–12 weeks; weight, ∼22 g). Mice were anesthetized with isoflurane (5% induction and 1%–2% maintenance) during injection. After 7 days, the Matrigel plugs were excised for immunohistochemical staining.

The effects of endothelial miR-186 on NSCLC vascularization and growth were investigated in a mouse flank tumor model as previously described. ^52^ Briefly, 1.8 × 10^6^ NCm- or miR-186m-transfected HDMECs together with 1.5 × 10^5^ NCI-H460 cells were suspended in 75 μL of phosphate-buffered saline and then injected subcutaneously into the right flank of NOD-SCID mice (n = 9 per group; age, 8–12 weeks; weight, ∼25 g). The mice were anesthetized with isoflurane (5% induction and 1%–2% maintenance) during injection. The volume of the developing tumors was quantified by measuring the two perpendicular diameters twice a week with a digital caliper and was calculated using the formula V = 1/2 (longer diameter) × (shorter diameter).^2^ On day 14, the tumors were excised for immunohistochemical staining.

### Immunohistochemistry

For the analysis of the microvessel density within the Matrigel plugs and tumor samples, 3-μm-thick sections were cut and stained with a rabbit anti-human CD31 antibody (1:150; Abcam, Cambridge, UK; ab32457) or a rat anti-mouse CD31 antibody (1:150; Dianova, Hamburg, Germany; DIA-310) followed by a goat-anti-rabbit Alexa Fluor 488-labeled secondary antibody (1:150; Life Technologies; Thermo Fisher Scientific; #A-11008) or a goat-anti-rat Alexa Fluor 555-labeled secondary antibody (1:150; Life Technologies; Thermo Fisher Scientific; #A-21434). The cell nuclei in the tissue sections were labeled with Hoechst 33342 (2 μg/mL; Sigma-Aldrich, Merck KGaA). After photographing the sections with a fluorescence microscope (BX-60; Olympus, Tokyo, Japan), the microvessel density was calculated by counting the number of CD31-positive microvessels in 10 fields of each section at 200× magnification and dividing by the area of each field.

To identify apoptotic tumor cells, sections were incubated with a rabbit anti-human cleaved caspase-3 antibody (1:150; Cell Signaling Technology, Frankfurt, Germany; #9661) followed by a biotin-conjugated goat anti-rabbit secondary antibody (1:150; Abcam; ab64256) and horseradish peroxidase-conjugated streptavidin (ready to use; Abcam). Afterward, the sections were sequentially exposed to 3-amino-9-ethylcarbazole substrate (AEC; Abcam) and Mayer’s hemalum solution (Merck KGaA). The sections were then analyzed under a BX-60 microscope (Olympus), and the percentage of cleaved caspase 3-positive tumor cells was quantified in 12 fields of each section at 400× magnification using the ImageJ software (NIH).

### Western blotting

Cells were first lysed with fresh lysis buffer consisting of 10 mM Tris (pH 7.5), 10 mM NaCl, 0.1 mM EDTA, 0.5% Triton X-100, 0.02% NaN_3_, phenylmethylsulfonylfluoride (1:500), protease inhibitor cocktail (1:100; Sigma-Aldrich), and phosphatase inhibitor cocktail (1:100; Sigma-Aldrich) for 10 min on ice. Then, the cell lysate was transferred to tubes and centrifuged at 4°C for 30 min at 13,000 × *g*. The supernatant was collected followed by the determination of protein concentrations using the Pierce BCA Protein Assay (Thermo Fisher Scientific) with BSA as a standard. Subsequently, the protein samples were separated by SDS-PAGE and electrotransferred onto a polyvinylidene difluoride membrane (Bio-Rad Laboratories, Munich, Germany). After blocking with Blotting-Grade Blocker (Bio-Rad Laboratories), the membrane was incubated with a rabbit anti-PKCα antibody (1:250; Cell Signaling Technology; #2056), a rabbit monoclonal anti-p-FAK antibody (1:250; Cell Signaling Technology; #8556), a rabbit polyclonal anti-FAK antibody (1:100; Cell Signaling Technology; #3285), a rabbit monoclonal anti-p-AKT antibody (1:300; Cell Signaling Technology; #4060), a rabbit anti-AKT antibody (1:300; Cell Signaling Technology; #4685), a mouse anti-p-ERK antibody (1:500; Abcam; ab50011), a rabbit anti-ERK antibody (1:500; Abcam; ab115799), a rabbit monoclonal anti-p-p38 antibody (1:250; Cell Signaling Technology; #4511), a rabbit polyclonal anti-p38 antibody (1:250; Cell Signaling Technology; #9212), or a mouse monoclonal anti-β-actin antibody (1:5,000; Sigma-Aldrich; A5441) followed by an anti-mouse (1:1,000; R&D Systems; HAF007) or anti-rabbit secondary antibody conjugated to horseradish peroxidase (1:1,000; R&D Systems; HAF008). The signal of these proteins was visualized using the Pierce ECL Western Blotting Substrate (Thermo Fisher Scientific) under a Chemocam device (Intas, Göttingen, Germany). The integrated intensity of each protein band was quantified using the ImageJ software (NIH).

### qRT-PCR

For the analysis of miRNA expression, total RNA extraction from ECs dissected by LCM and cultured ECs was performed using the miRNeasy FFPE Kit (Qiagen) and miRNeasy mini kit (Qiagen), respectively. The concentration of the eluted RNA was measured with the DeNovix DS-11 spectrophotometer (Biozym Scientific, Hessisch Oldendorf, Germany), and RNA integrity was verified with the Agilent Bioanalyzer using the Nano RNA Kit (Agilent Technologies, Santa Clara, CA, USA). Then, 1 μg of RNA was converted to cDNA using the miScript Reverse Transcription Kit (Qiagen). Noteworthy, after reverse transcription, cDNA of dissected ECs by LCM was further amplified using the miScript PreAMP PCR Kit (Qiagen). For -primary miRNA (pri-miRNA) and gene detection, total RNA was extracted from cultured cells using the RNeasy mini kit (Qiagen) and converted to cDNA using the QuantiTect Reverse Transcription Kit (Qiagen). The further qRT-PCR assay was performed with 100 ng of cDNA using the miScript and QuantiTect SYBR Green PCR Kit (Qiagen) for the detection of miRNA and mRNA, respectively. A total of 40 cycles were performed in the Bio-Rad MiniOpticon Real-Time PCR System (Bio-Rad Laboratories), and the cycling parameters were as follows: initial activation at 95°C for 15 min, denaturation at 94°C for 15 s, annealing at 55°C for 30 s, and extension at 70°C for 34 s (miScript SYBR Green PCR Kit) or extension at 72°C for 30 s (QuantiTect SYBR Green PCR Kit). The relative expression levels of miRNA, pri-miRNA, and genes were calculated using the 2^−ΔΔCt^ method with small nucleolar RNA RNU6B (U6) and glyceraldehyde-3-phosphate dehydrogenase (GAPDH) as endogenous control, respectively. The miScript primer assays for Hs_miR-186_1 and Hs_RNU6-2_11 were purchased from Qiagen. The gene-specific primers were purchased from Invitrogen (Thermo Fisher Scientific) and their sequences are listed in Table S2.

### Plasmid construction

According to the TargetScan 7.1 algorithm, there are four putative binding sites of miR-186 in the 3′ UTR of PRKCA. Due to the distant distribution of these binding sites, two parts of the 3′ UTR sequence of PRKCA were separately amplified from the genomic DNA of HEK293T cells and subcloned into the region directly after the stop codon of luciferase gene in a firefly luciferase reporter vector. One part contained one putative binding site of miR-186, while the other one consisted of three putative binding sites. Mutant 3′ UTR was obtained from the cloned PRKCA-3′ UTR using the QuikChange Lightning Site-Directed Mutagenesis Kit (Agilent Technologies, Shanghai, China). Both the wild-type and mutant 3′ UTR sequences were confirmed by sequencing (Invitrogen; Thermo Fisher Scientific). The sequences of the used primers are listed in Table S3.

### Luciferase reporter assay

To verify whether PRKCA is a direct target of miR-186, 10 ng of firefly luciferase plasmid (with or without PRKCA-3′ UTR), 15 ng of Renilla luciferase plasmid, and 100 nM miR-186m or NCm were co-transfected into HEK293T cells in 96-well plates using the Lipofectamine 2000 transfection reagent (Thermo Fisher Scientific). After incubation for 48 h, the cells were lysed and firefly and Renilla luciferase activities of the cell lysates were determined using the Dual-Luciferase Reporter Assay Kit (GeneCopoeia, Heidelberg, Germany) in a Tecan Infinite M200 PRO luminometer (Crailsheim, Germany). Luciferase activity was calculated by dividing the firefly luciferase signal of each sample by its corresponding Renilla luciferase signal.

### Statistics

Statistical analyses were performed using GraphPad Prism 9.0.0. The data were first tested for normal distribution and equal variance. In case of parametric data, differences between two groups were analyzed by a paired Student’s t test (for the analysis of patient samples) or unpaired Student’s t test (for other analyses). In the case of non-parametric data, a Mann-Whitney U test was used (for the analysis of western blot data). One-way ANOVA followed by the Tukey’s multiple comparisons test was used for statistical comparisons between multiple groups. All data were expressed as means ± SEM. Statistical significance was accepted for p values <0.05.

## Data Availability

All data generated or analyzed during this study are included in this published article and its supplementary information files.

## Supplemental information

Document S1. Figures S1–S 5 and Tables S1–S3

Document S2. Article plus supplemental information
