# Supplementary material for: Hypoxia-induced downregulation of microRNA-186-5p in endothelial cells promotes non-small cell lung cancer angiogenesis by upregulating protein kinase C alpha
Source: Mol Ther Nucleic Acids. 2023 Jan 27;31:421–36. doi: 10.1016/j.omtn.2023.01.015 (PMC9945639; doi:10.1016/j.omtn.2023.01.015)
Supplement: Document S1. Figures S1–S — 5 and Tables S1–S3 [file mmc1.pdf]

OMTN, Volume *31*

Supplemental information

Hypoxia-induced downregulation of microRNA-186-5p in endothelial cells promotes non-small cell lung cancer angiogenesis by upregulating protein kinase C alpha

Vivien Becker, Xu Yuan, Anne S. Boewe, Emmanuel Ampofo, Elke Ebert, Johannes Hohneck, Rainer M. Bohle, Eckart Meese, Yingjun Zhao, Michael D. Menger, Matthias W. Laschke, and Yuan Gu

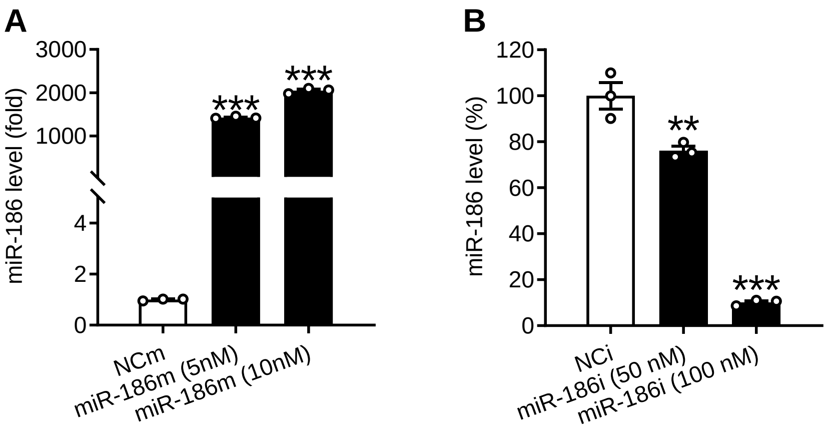

Figure S1. Transfection efficiency of miR-186m and miR-186i

(A) miR-186 level (fold of NCm) in HDMECs that were transfected with NCm, 5 nM or 10 nM miR-186m, as assessed by qRT-PCR (n = 3). (B) miR-186 level (percentage of NCi) in HDMECs that were transfected with NCi, 50 nM or 100 nM miR-186i, as assessed by qRT-PCR (n = 3). Means ± SEM. **P < 0.01, ***P < 0.001 vs. NCm or NCi (one-way ANOVA with Tukey's multiple comparisons test).

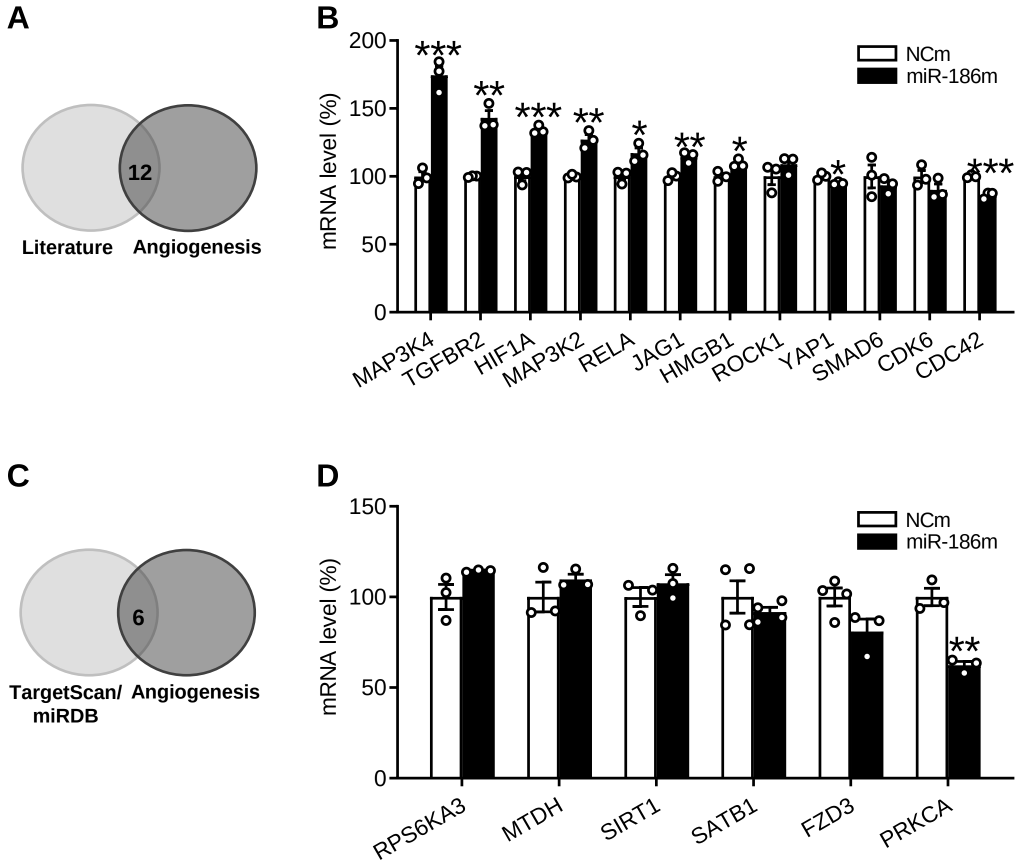

Figure S2. Expression of predicted and validated human targets of miR-186 in HDMECs

(A) Venn diagram displaying the identification of 12 validated human targets of miR-186 that are known to regulate angiogenesis. (B) mRNA levels (percentage of NCm) of the validated miR-186 targets in NCm- or miR-186m-transfected HDMECs, as assessed by qRT-PCR (n = 3). (C) Venn diagram displaying the prediction of 6 human miR-186 target genes that are involved in angiogenesis. (D) mRNA levels (percentage of NCm) of the putative miR-186 targets in NCm- or miR-186m-transfected HDMECs, as assessed by qRT-PCR (n = 3). Means ± SEM. *P < 0.05, **P < 0.01, ***P < 0.001 vs. NCm (unpaired Student’s t test).

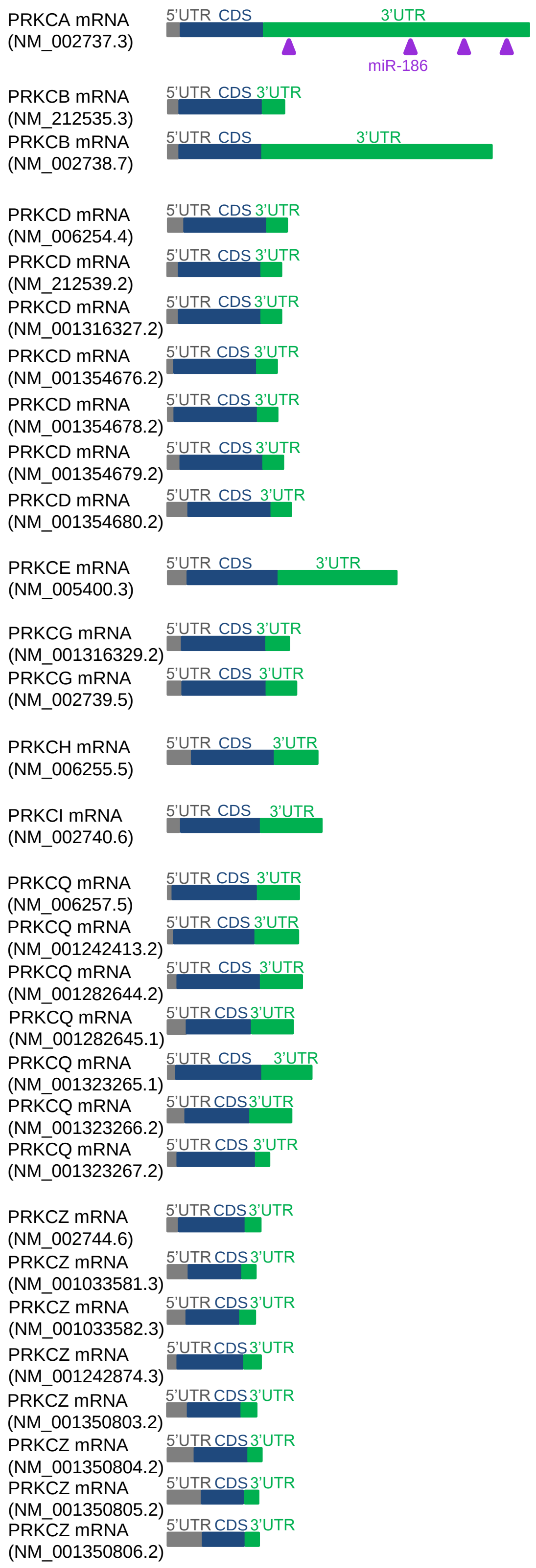

Figure S3. Putative binding sites of miR-186 within the 3'UTR of the human PRKC family

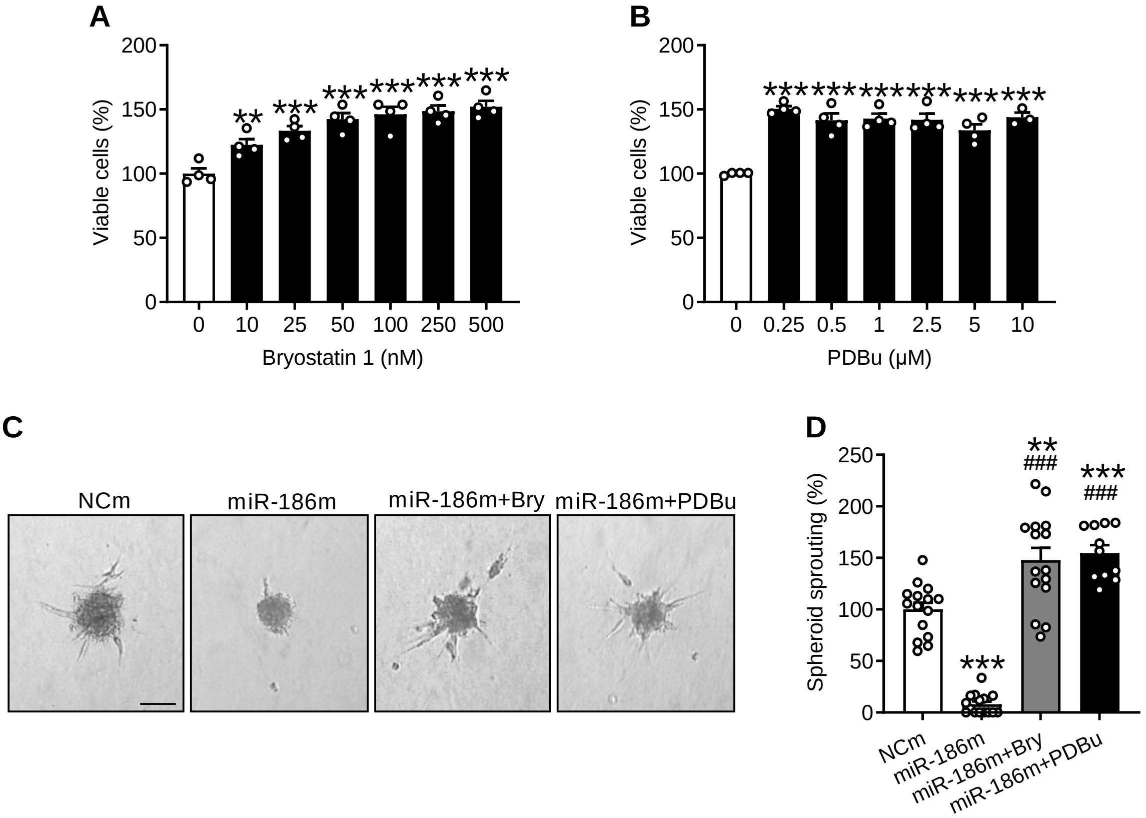

Figure S4. Effects of bryostatin 1 and PDBu on miR-186m-suppressed EC spheroid sprouting

(A and B) Number of viable HDMECs (percentage of 0 µM), as assessed by WST-1 assay (n = 4). The cells were exposed for 72 h to a serial dilution of bryostatin 1 (A) or PDBu (B). (C) Phase-contrast microscopy images of HDMEC spheroids. The spheroids of NCm- or miR-186m-transfected HDMECs were treated with or without 100 nM bryostatin 1 (Bry) and 1 µM PDBu for 24 h. Scale bar, 85 µm. (D) Spheroid sprouting (percentage of NCm) of HDMECs that were treated as described in (C), as assessed by spheroid sprouting assay (n = 11-15). Means ± SEM. **P < 0.01, ***P < 0.001 vs. 0 nM, 0 µM, NCm (one-way ANOVA with Tukey's multiple comparisons test). ###P < 0.001 vs. miR-186m (D, one-way ANOVA with Tukey's multiple comparisons test).

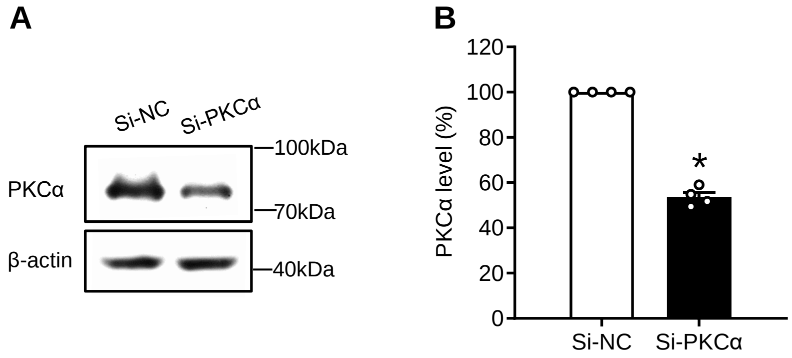

Figure S5. Transfection efficiency of si-PKCα

(A) Representative western blots of PKCα and β-actin expression in si-NC- or si-PKCα-transfected HDMECs. (B) Expression level (percentage of si-NC) of PKCα in HDMECs that were transfected with si-NC or si-PKCα, as assessed by western blotting (n = 4 independent experiments). Means ± SEM. *P < 0.05 vs. si-NC (Mann-Whitney U test).

Table S1. Pathological characteristics of NSCLC patients

Patient	Gender	Age	Tumor size	Tumor grade	Lymph node metastasis	Lymphatic invasion	Vascular invasion
1	female	52	pT2a	G2	pN0	no	yes
2	male	65	pT2a	G3	pN2	yes	yes
3	male	86	pT2a	G3	pN0	yes	no
4	female	66	PT3	G3	pN1	yes	yes
5	male	73	pT2b	G3	pN2	yes	yes
6	male	52	pT1a	G3	pN2	no	yes
7	male	70	pT2a	G2	pN0	yes	yes
8	male	70	pT3	G2	pN0	no	no
9	male	73	pT1b	G3	pN0	no	no
10	male	71	pT2a	G3	pNX	n.a.	n.a.
11	male	75	pT3	G2	pN2	no	no

n.a.: not available.

Table S2. Forward and reverse primers for quantitative real-time PCR analyses

	Forward primer (5'→3')	Reverse primer (5'→3')
**pri-miR-186**	ACCAGGTATATGGCACAGCAA	CCTGAAGCATGTGAATGTCAAC
**CDC42**	CCATCGGAATATGTACCGACTG	CTCAGCGGTCGTAATCTGTCA
**CDK6**	CCAGATGGCTCTAACCTCAGT	AACTTCCACGAAAAAGAGGCTT
**FZD3**	GTTCATGGGGCATATAGGTGG	GCTGCTGTCTGTTGGTCATAA
**GAPDH**	ATGGGTGTGAACCATGAGAAGTA	GGCAGTGATGGCATGGAC
**HIF1A**	GAACGTCGAAAAGAAAAGTCTCG	CCTTATCAAGATGCGAACTCACA
**HMGB1**	GCGGACAAGGCCCGTTA	AGAGGAAGAAGGCCGAAGGA
**JAG1**	GTCCATGCAGAACGTGAACG	GCGGGACTGATACTCCTTGA
**MAP3K2**	CCCCAGGTTACATTCCAGATGA	GCATTCGTGATTTTGGATAGCTC
**MAP3K4**	GCAAAGCCATCCCAAGTT	GTGCCTCTATGTTCATTCTGTT
**MTDH**	CTCGGGCTGCTGCTGCTGTT	CAGCAAGGCCAGGTCGTCGG
**PRKCA**	GTCCACAAGAGGTGCCATGAA	AAGGTGGGGCTTCCGTAAGT
**RELA**	ATGTGGAGATCATTGAGCAGC	CCTGGTCCTGTGTAGCCATT
**ROCK1**	AACATGCTGCTGGATAAATCTGG	TGTATCACATCGTACCATGCCT
**RPS6KA3**	GTGGCAGAAGATGGCTGTG	TGGGTTAATCTCCTCCTCTCC
**SATB1**	CCAGGTTGGAAAGTGGAATCC	GGGGCAACTGTGTAACTGAAT
**SIRT1**	TAGCCTTGTCAGATAAGGAAGGA	ACAGCTTCACAGTCAACTTTGT
**SMAD6**	CCTCCCTACTCTCGGCTGTC	GGTAGCCTCCGTTTCAGTGTA
**TGFBR2**	AAGATGACCGCTCTGACATCA	CTTATAGACCTCAGCAAAGCGAC
**YAP1**	TAGCCCTGCGTAGCCAGTTA	TCATGCTTAGTCCACTGTCTGT

Table S3. Forward and reverse primers for plasmid construction

	Forward primer (5’→3’)	Reverse primer (5’→3’)
**PRKCA-3’UTR WT1**	GGGGTACCAAGCCAAGAGAGTGAGCAGG	CCGCTCGAGAGGGAAAGCACAGAAGCAAG
**PRKCA-3’UTR WT2**	GGGGTACCCTGAGGATGAATGGAGAAGT	CCGCTCGAGACAGAAGGGATGTGAGGTTA
**PRKCA-3’UTR WT2 MT1**	CACCATTTCCTGTCCTAGAGTAACCCGACAAGGGTAGGAGT	CCTTGTCGGGTTACTCTAGGACAGGAAATGGTGTGATGTGCA
**PRKCA-3’UTR WT2 MT2**	ACTTTAATAATACCTAGAGCAAAATGAGTTTTTAGAACAAAGCAA	AAAACTCATTTTGCTCTAGGTATTATTAAAGTTCTCTATAAAAAA
**PRKCA-3’UTR WT2 MT3**	CTGGAAGAATATCCCTAGAGTCTTGAAGGGTTGGTTTACTAGAAT	CAACCCTTCAAGACTCTAGGGATATTCTTCCAGGAAGAATATCAA
